# The Origin of Mitochondria-Specific Outer Membrane β-Barrels from an Ancestral Bacterial Fragment

**DOI:** 10.1093/gbe/evy216

**Published:** 2018-09-28

**Authors:** Joana Pereira, Andrei N Lupas

**Affiliations:** Department of Protein Evolution, Max-Planck-Institute for Developmental Biology, Tübingen, Germany

**Keywords:** motif amplification, protein evolution, remote homology, mitochondria, outer membrane

## Abstract

Outer membrane β-barrels (OMBBs) are toroidal arrays of antiparallel β-strands that span the outer membrane of Gram-negative bacteria and eukaryotic organelles. Although homologous, most families of bacterial OMBBs evolved through the independent amplification of an ancestral ββ-hairpin. In mitochondria, one family (SAM50) has a clear bacterial ancestry; the origin of the other family, consisting of 19-stranded OMBBs found only in mitochondria (MOMBBs), is substantially unclear. In a large-scale comparison of mitochondrial and bacterial OMBBs, we find evidence that the common ancestor of all MOMBBs emerged by the amplification of a double ββ-hairpin of bacterial origin, probably at the time of the Last Eukaryotic Common Ancestor. Thus, MOMBBs are indeed descended from bacterial OMBBs, but their fold formed independently in the proto-mitochondria, possibly in response to the need for a general-purpose polypeptide importer. This occurred by a process of amplification, despite the final fold having a prime number of strands.

## Introduction

Amplification of subdomain-sized fragments is a dominant phenomenon in the evolution of protein folds, resulting in repetitive proteins that adopt a pseudosymmetrical fold (Andrade et al. 2001; Söding and Lupas 2003; Alva and Lupas 2018). One of these is the outer membrane β-barrel (OMBB), a closed antiparallel β-sheet whose strands traverse the outer membrane in Gram-negative bacteria, but also mitochondria, mitochondria-related organelles and plastids (Duy et al. 2007; Remmert et al. 2010; Zeth and Thein 2010; Chaturvedi and Mahalakshmi 2017). OMBBs preform a wide array of functions, from solute transport to membrane protein assembly, and are composed of a variable number of β-strands.

**Fig. 1.**
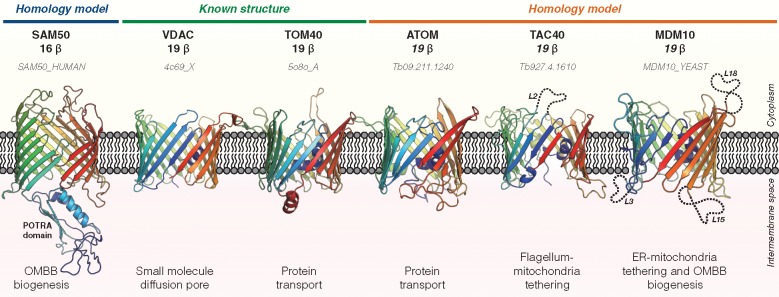
—Three-dimensional structure and biological function of mitochondrial outer membrane β-barrels (MOMBBs). Six outer membrane β-barrel subfamilies have been described so far in mitochondria: The 16-stranded SAM50/TOB55, which belongs to the OMP85 family of bacterial OMBBs and is involved in the biogenesis and membrane insertion of OMBBs (Kozjak et al. 2003), and the five members of the 19-stranded OMBB family unique to mitochondria (MOMBBs) TOM40, VDAC, MDM10, ATOM, and TAC40 (Pusnik et al. 2011; Bay et al. 2012; Zarsky et al. 2012; Flinner et al. 2013; Schnarwiler et al. 2014). For those whose three-dimensional structure is known (VDAC and TOM40), the experimental structure is shown. For those whose structure was not yet experimentally determined, homology models are shown only for illustrative purposes. For that, the best templates for the reference sequences were identified with HHPred (Zimmermann et al. 2018) over the PDB70 (as of May 2018; supplementary table 1, Supplementary Material online) and the models built with SWISS-MODEL (Biasini et al. 2014) after target-template alignment with PROMALS3D (Pei and Grishin 2014). Long loops are shown in dashed cartoon lines for clarity and named L*x*, where *x* refers to the ranking of the loop in the predicted structure.

Gram-negative OMBBs have an even number of β-strands between 8 and 26 (Koebnik et al. 2000; Chaturvedi and Mahalakshmi 2017). Internal sequence symmetry suggests that the major families of OMBBs arose independently through the amplification of a homologous pool of ancestral ββ-hairpins (Remmert et al. 2010). While bacterial outer membranes contain many families of OMBBs, mitochondrial ones contain only two (fig. 1). One, formed by the 16-stranded SAM50/TOB55, clearly belongs to the OMP85 family of bacterial OMBBs (Kozjak et al. 2003). The other, comprising the 19-stranded TOM40 and VDAC (Bay et al. 2012), is found only in mitochondria and its origins are as yet unclear (Cavalier-Smith 2006; Zeth and Thein 2010). In the following we will refer to this family as mitochondria-only OMBBs (MOMBBs). In addition to TOM40 and VDAC, which are present in almost all lineages of eukaryotes (supplementary fig. 1, Supplementary Material online), this family also contains three lineage-specific members: MDM10 from fungi and amoebozoa (Flinner et al. 2013); and TAC40 and ATOM, both from trypanosoma (Pusnik et al. 2011; Zarsky et al. 2012; Schnarwiler et al. 2014). In order to shed light on the origins of this family, we carried out a broad survey of OMBBs in mitochondria and bacteria.

## Results and Discussion

Using PSI-BLAST, we screened the nonredundant protein database at NCBI for homologs of known MOMBBs (see Materials and Methods). We retrieved a set of 1,394 sequences, which illustrate the distribution of the five MOMBB subfamilies in the major eukaryotic lineages (supplementary fig. 1, Supplementary Material online). No bacterial matches were found for VDAC, TOM40, TAC40, and MDM10, but searches with ATOM resulted in four incomplete matches to a family of uncharacterized bacterial OMBBs. This family is putatively 12-stranded and distantly related to FapF, an OMBB involved in the secretion of amyloid subunits during biofilm formation (Rouse et al. 2017). These searches did not result in any, even marginally significant matches to SAM50.

**Fig. 2.**
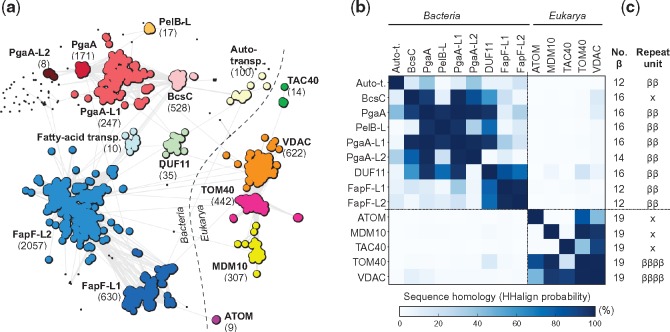
—Classification and HMM-comparison of bacterial and mitochondrial OMBBs (MOMBBs). (*a*) Cluster map of a total of 5277 sequences collected for each MOMBB family and bacterial FapF-like and PgaA-like OMBBs. Clustering was performed with CLANS in 2D until equilibrium at a BLASTp *P*-value of 1.0. Connections represent similarities up to a *P*-value of 10^−3^ (darker means more similar). Black points represent sequences that do not connect to any cluster at *P*-values <10^−4^. The number of sequences in each cluster is shown within brackets. Clusters composed solely by hypothetical and nonannotated sequences but with significant homology to a known protein family, as detected with HHPred and PSI-BLAST searches, are referred by the name of the homologous family followed by L*x*, where *x* represents the number of the cluster. The taxonomic distribution of the collected sequences is illustrated in supplementary figures 1 and 2, Supplementary Material online. A total of five eukaryotic and eight bacterial clusters were obtained. VDAC, TOM40, and MDM10 form a highly connected supercluster, which connects only marginally with bacterial OMBBs. TAC40 connects to VDAC, but ATOM does not connect to any cluster at a *P*-value <10^−3^. (*b*) Sequence homology matrix of OMBB clusters as measured by the hhalign probability of the alignment of their HMM-profiles. Those corresponding to the “Fatty-acid transporters” cluster were not included due to the high level of fragmentation of the sequences composing it. Bacterial and eukaryotic OMBBs define two different regions and all MOMBBs find only marginal matches to bacterial OMBBs, especially BcsC and PgaA, suggesting that all MOMBBs are monophyletic and share only local sequence similarity to OMBBs. (*c*) Strand composition predicted with Quick2D and repeat units identified with HHrepID for the HMM-profile consensus sequence. All MOMBBs are predicted to have a 19-stranded topology; additionally, VDAC and TOM40 show a repetitive sequence. No bacterial OMBB shows the same topology and repetition pattern. ββ: ββ-hairpin; ββββ: double ββ-hairpin; x: none or not clear.

When clustered based on their sequence similarity (fig. 2*a*), VDAC, TOM40, and MDM10 form a highly connected supercluster to which TAC40 links via VDAC. ATOM sequences connect only distantly to other MOMBBs and cluster closer to bacterial FapF-like OMBBs. In HMM-profile searches, all five MOMBBs make statistically significant, full-length matches to either VDAC or TOM40, but not to bacterial OMBBs (fig. 2*b* and supplementary fig. 3, Supplementary Material online). Only local matches (with a coverage of 20–40%) are found between mitochondrial and bacterial OMBBs, especially VDAC and TOM40. These results support the notion that all MOMBBs, including ATOM (Zarsky et al. 2012), are monophyletic and share local sequence similarity to bacterial OMBBs.

**Fig. 3.**
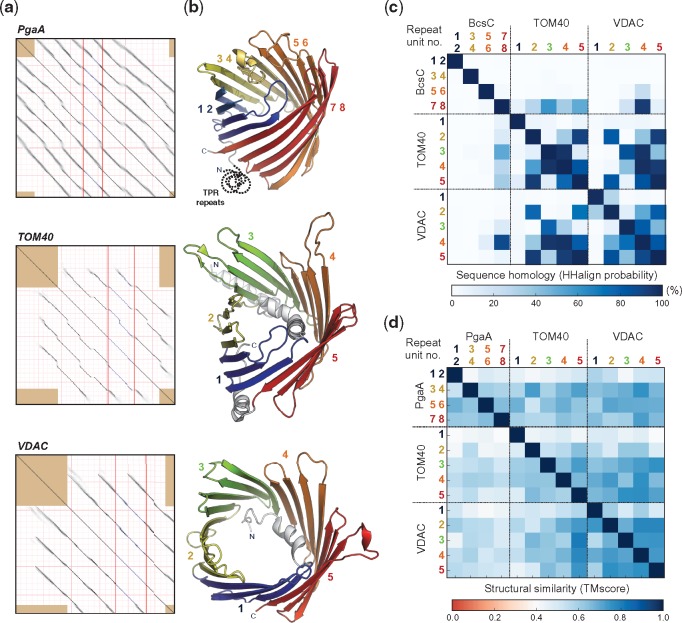
—The repetitive nature of VDAC, TOM40, and BcsC/PgaA OMBBs. (*a*) Self-comparison dot plot of the PgaA, TOM40, and VDAC HMM-consensus sequence generated by HHrepID. The presence of diagonal lines indicates a repetitive sequence. Repeat families were identified at a *P*-value threshold of 10^−1^. For VDAC and TOM40, the full consensus sequence included the N-terminal helix, colored grey in (*b*). Eight sequence repeats were identified in PgaA, whereas only five were found in VDAC and TOM40. (*b*) Three-dimensional mapping of the identified repeats on their reference three-dimensional structures (PgaA: 4y25_A; TOM40: 5o8o_A; VDAC: 4c69_X). The sequence repeats in PgaA correspond to single ββ-hairpins, while those of VDAC and TOM40 correspond to two ββ-hairpins. (*c*) Sequence homology matrix, measured as the hhalign probability of the alignment of the HMM-profiles built for VDAC, TOM40, and BcsC (as mapped over PgaA) double ββ-hairpins. The repeats in VDAC and TOM40 find significant matches only with the last C-terminal double ββ-hairpin of BcsC, with the best match found between this and the fourth repeat of VDAC. (*d*) Structural similarity matrix, measured as the TMscore from structural alignments with TMalign, of VDAC, TOM40, and PgaA double ββ-hairpins. A TMscore below 0.3 indicates random structural similarity while values above 0.5 suggests that both structures assume the same fold. A TMscore of 1.0 denotes a perfect match between the two structures. The predominantly blue matrix suggests that, despite their low sequence similarity, all double ββ-hairpins are structurally conserved and thus the high level of similarity between the fourth repeat of VDAC and the C-terminal strands of BcsC is not the result of structural constraints.

**Fig. 4.**
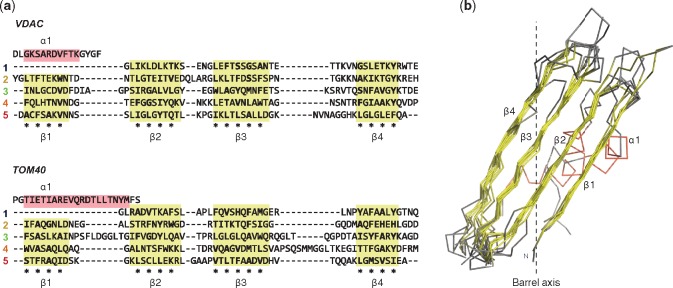
—Sequence and structural conservation of the repeat units from VDAC and TOM40. (*a*) Structure-based sequence alignment of the repeats identified in the HMM-consensus sequence of VDAC and TOM40 with HHrepID. Repeats were mapped onto their reference structure (TOM40: 5o8o_A; VDAC: 4c69_X), and (*b*) the structural superposition of the corresponding double ββ-hairpins was carried out with TMalign and manually adjusted using UCSF Chimera (Pettersen et al. 2004) without considering the N-terminal helix and the loop regions. In (*a*), the position and boundaries of the helices and strands, as of their reference structure, are shaded red and yellow, respectively; asterisks mark strand positions facing the outside of the barrel, with those in bold depicting hydrophobic, aromatic or small residues. Each sequence repeat is composed of two ββ-hairpins, with the exception of the first repeat, where the first strand appears to have been changed to an α-helix. All these double ββ-hairpins have a closely matching structure. In (*b*), a dashed line represents the transmembrane axis of the reference barrels, highlighting the strand tilt with respect to the membrane.

Previous analysis has shown that most families of OMBBs have a clear repeat signature in their sequences, in which the repeating unit coincides with the structural ββ-hairpin repeat (Remmert et al. 2010). In MOMBBs, only VDAC and TOM40 have a detectable sequence repeat (figs. 2*c* and 3*a*; Remmert et al. 2010; Zeth and Thein 2010), and here the repeating unit is composed of two ββ-hairpins (figs. 3*b* and 4). The double ββ-hairpins from VDAC and TOM40 have closely matching structures (fig. 4*b*) and, while it may seem counterintuitive that a fold obtained by repetition of one structural unit could have a prime number of strands, the sequence alignment of the repeats shows that the first one lacks the first strand, which may have been converted to a helix, in order to generate a plug (fig. 4*a*).

**Fig. 5.**
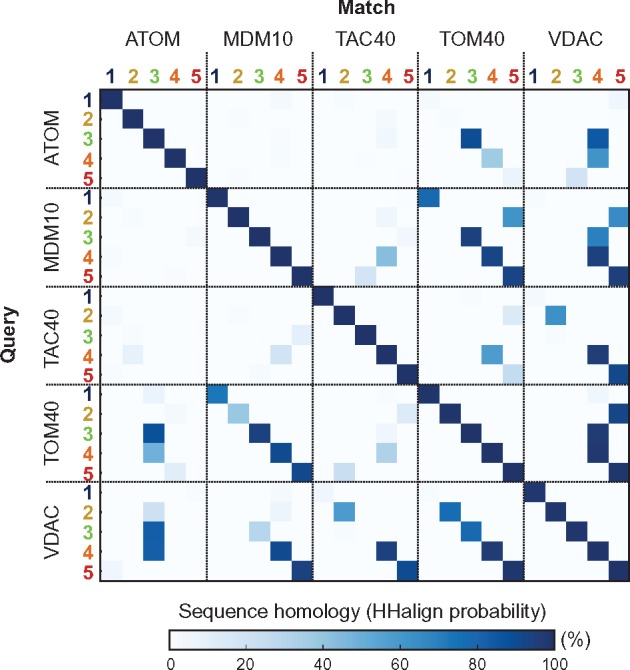
—Sequence homology matrix of double ββ-hairpins from MOMBBs, measured as the hhalign probability of the alignment of their HMM-profiles built with hhmake. For clarity, only the top match of a given query double ββ-hairpin in each of the MOMBB subfamilies is shown. Almost invariably, significant matches for repeat *n* of one MOMBB has its best match in repeat *n′* of another MOMBB, suggesting that all 19-stranded MOMBBs diverged from a fully amplified ancestor and were not amplified individually.

To test whether MOMBBs may have been amplified independently from the same structural unit, we compared each repeat with all the others in our set of MOMBBs. Almost invariably, where significant matches were obtained, repeat *n* of one MOMBB had its best match in repeat *n′* of another MOMBB (figs. 3*c* and 5). From this we conclude that 19-stranded MOMBBs diverged from a fully amplified ancestor, rather than being amplified individually.

Next, we searched for clues to the origin of the fragment from which MOMBBs were amplified. Because VDAC and TOM40 are the only MOMBBs that still show recognizable internal sequence symmetry, we focused the search on their repeats. Searches with HHsearch over the PFAM (Finn et al. 2016), TIGRFAMs (Haft et al. 2003), COG (Galperin et al. 2015), and NCBI’s Conserved Domains (CD; Marchler-Bauer et al. 2015) databases identified numerous matches to OMBB families at a significance >50% (supplementary table 2, Supplementary Material online). These connect MOMBBs to OMBBs that are mostly involved in small molecule transport in a wide range of Gram-negative bacteria, especially proteobacteria. Where detectable, the repeats of these OMBBs correspond to single ββ-hairpins, not double ones as in VDAC and TOM40.

Although MOMBBs and OMBBs share a conserved C-terminal β-signal for membrane insertion (Kutik et al. 2008; Walther et al. 2009), and indeed the majority of the matches covered the C-terminal hairpin of the OMBBs, they were all obtained with the fourth repeat from VDAC, not the fifth, C-terminal one (supplementary table 2, Supplementary Material online). The best match obtained was to the last three strands of the BcsC family of sugar transporters (Whitney and Howell 2013), which is also the only matched OMBB family found in α-proteobacteria. This seemed particularly attractive, since mitochondria are thought to have descended from α-proteobacteria (Andersson et al. 1998; Roger et al. 2017). BcsC proteins share local and global sequence similarity with several families of transporters, including PgaA and FapF-like proteins (fig. 2). While BcsC does not show detectable sequence repeats, PgaA has a clear ββ-hairpin repeat and is also the only one of known structure (fig. 3*a* and *b*). Using it as a structural prototype for the BcsC family, we find in comparisons to VDAC and TOM40 that all double ββ-hairpins have closely matching structures (fig. 3*c* and *d*). This suggests that the high level of similarity between the fourth repeat of VDAC and the C-terminal strands of BcsC is not the result of structural constraints.

## Conclusions

In conclusion, our analysis confirms the monophyletic relationship of VDAC and TOM40 (Bay et al. 2012), and extends it to all MOMBBs including ATOM, for which our results confirm that it is a distant form of TOM40 and did not evolve independently from a bacterial OMBB (Zarsky et al. 2012). As MOMBBs and OMBBs match in sequence only locally, and VDAC and TOM40 were probably part of the Last Eukaryotic Common Ancestor (LECA) proteome, it seems likely that the ancestor of all MOMBBs emerged in the proto-mitochondrion and was not acquired from the proteobacterial endosymbiont. Instead, it evolved independently by the amplification of a double ββ-hairpin related to those of OMBBs. The evolution of a new outer-membrane pore may have been driven by the need for a general-purpose polypeptide importer, a function for which there are no prototypes in the bacterial outer membrane. This need would have arisen in the early stages of endosymbiosis, after an increasing number of genes were transferred from the symbiont to the host nucleus, requiring it to reimport the encoded proteins. If this scenario is correct, then the ancestral function of MOMBBs would have been polypeptide import, possibly facilitated by sensitivity to an electrochemical gradient. The electrochemically gated diffusion of small molecules mediated by VDAC would then have represented a subsequent evolutionary development. The de novo evolution of a new pore implies that it was initially independent of signal sequences, which would have gradually evolved with the acquisition of further TOM proteins to the import machinery (Garg et al. 2015).

As the best match between MOMBBs and OMBBs covers the C-terminal strands of BcsC and this family occurs in α-proteobacteria, it seems attractive to propose that the last four strands of a proteobacterial transporter related to BcsC were amplified during the transition from a free-living organism to an endosymbiotic organelle at the time of the LECA. The amplification of these strands would have been particularly advantageous as they already include the appropriate sequence signal for targeting and assembly into the membrane.

The amplification of the 4-stranded fragment would have yielded a 20-stranded barrel, yet MOMBBs have 19 strands. Given the size of the N-terminal α-helix present in all MOMBBs (fig. 4), it is possible that this arose from the N-terminal strand, driven by the need to gate the newly evolved pore. This resulted in the present-day MOMBB architecture of a 19-stranded barrel surrounding an α-helical plug, which is an important determinant in the sensitivity of MOMBBs to electrochemical gradients (Tornroth-Horsefield and Neutze 2008). The 20-stranded barrel at the origin of MOMBBs would represent a fold not yet identified in any kingdom of life (Chaturvedi and Mahalakshmi 2017). While substantiating that MOMBBs descended from bacterial OMBBs, but their fold formed independently in the proto-mitochondria, our results also highlight the role of motif amplification in the de novo emergence of new forms for established protein architectures.

## Materials and Methods

### Assembly of the MOMBB and OMBB Sequence Set

We assembled our set of MOMBB and OMBB sequences by preforming four rounds of PSI-BLAST searches using the MPI Bioinformatics Toolkit (Zimmermann et al. 2018). Searches for MOMBB sequences were preformed over the *nr* database (as of May 2018) using the reference sequences of TOM40, VDAC, MDM10, ATOM, and TAC40 (fig. 1) while searches for FapF-like and BcsC-like OMBBs were preformed over the bacterial part of *nr* (*nr_bac*) (as of May 2018) using the sequences of the barrel regions identified in the sequences of *Rhodanobacter* sp*. Soil772* FapF-like (UniprotKB: A0A0Q9P8F2), *Pseudomonas* sp*. UK4* FapF (UniprotKB: C4IN73) and *Escherichiacoli* BcsC (UniprotKB: P37650) and PgaA (UniprotKB: P69434). In order to identify these barrel regions, we searched for reference structures for these sequences on the PDB70 and SCOPe databases (as of May 2018) with HHpred, without scoring for secondary structure, and predicted their secondary structure content with Quick2D (Alva et al. 2016). In both cases, the parameters were set to default.

### Classification and HMM-Comparison of MOMBB and OMBB Sequences

In order to classify the barrel sequences in our set, we first filtered them to a maximum sequence identity of 80% with MMseqs2 (Steinegger and Söding 2017) using a minimum alignment coverage of 0.0 and the normal clustering mode. The resulting sequences were then clustered with CLANS (Frickey and Lupas 2004) based on their BLASTp pairwise *P*-values computed using the BLOSUM62 scoring matrix. Clustering was performed until equilibrium at a BLASTp *P*-value of 1.0 and clusters identified manually at a *P*-value of 10^−3^.

HMM-comparisons of the obtained clusters were preformed by building and aligning their HHM-profiles. For that, the sequences in each major cluster were aligned with PROMALS3D (Pei and Grishin 2014) and the resulting alignments processed with trimAl (Capella-Gutierrez et al. 2009) by removing columns where >85% of the positions represent a gap (gap score of 0.15) and sequences that only overlap with <50% of the columns populated by 80% or more of the other sequences. These alignments were used to build HMM-profiles with hhmake which were further aligned with hhalign (Söding 2005). HMM-profile building and alignment were carried out using default parameters without secondary structure scoring.

### Identification and Comparison of Sequence Repeats

The repetitive nature of the HMM-consensus sequences was predicted with HHrepID (Biegert and Söding 2008; Zimmermann et al. 2018), using default parameters without the generation of a new multiple sequence alignment, and their secondary structure content predicted with Quick2D as described above. By extracting their corresponding regions in the alignments of the various barrels, we built HMM-profiles as described above for each of the repeats identified. The regions in MDM10, TAC40, and ATOM were assigned by mapping them to the VDAC and TOM40 consensus sequences, and those in BcsC by mapping to PgaA. To test the independent amplification of MOMBBs and OMBBs, the resulting HMM-profiles were aligned with hhalign, as described above, and the corresponding double ββ-hairpins structurally compared by structural alignment with TMalign (Zhang and Skolnick 2005).

### Identification of Bacterial OMBBs Matching MOMBB Repeats

To investigate the origins of the double ββ-hairpins from the ancestor of all MOMBBs, the HMM-consensus sequence of the double ββ-hairpins from VDAC and TOM40 were used for searches over the PFAM, TIGRFAM, CD, and COG databases (as of August 2018) with HHPred, without scoring for secondary structure. The secondary structure content and the repetitive nature of the protein families matched in the searches were predicted, respectively, with Quick2D and HHrepID as described above. The taxonomic distribution of these families was retrieved from PFAM and eggNOG (Huerta-Cepas et al. 2016) as of August 2018.

## Supplementary Material

Supplementary Data
