# Supplementary material for: The Origin of Mitochondria-Specific Outer Membrane β-Barrels from an Ancestral Bacterial Fragment
Source: Genome Biol Evol. 2018 Sep 28;10(10):2759–65. doi: 10.1093/gbe/evy216 (PMC6193526; doi:10.1093/gbe/evy216)
Supplement: Supplementary Data [file evy216_supp.docx]

**SUPPLEMENTARY FIGURES**

**
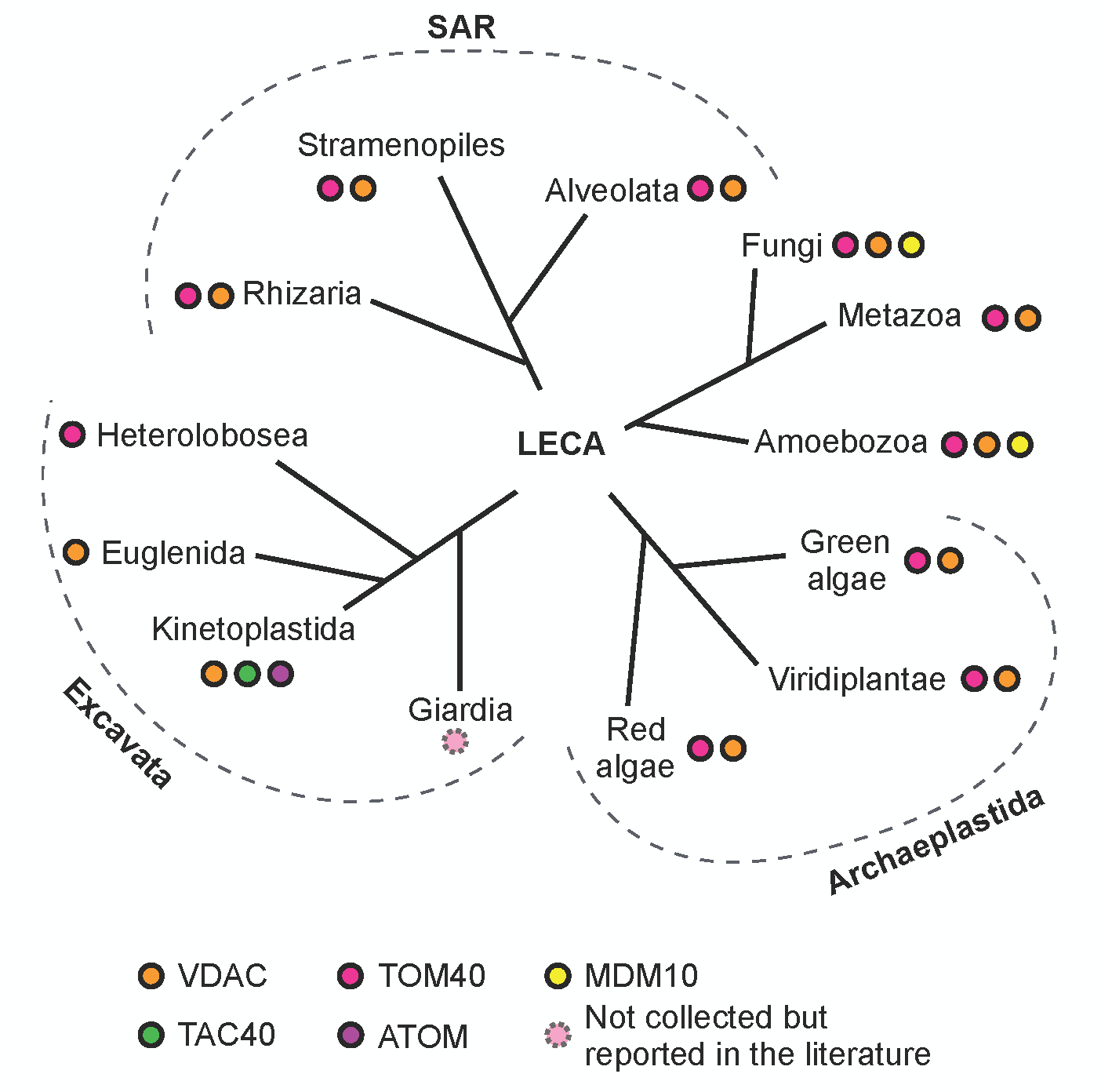
**

Supplementary figure 1. Taxonomic distribution of collected mitochondria-only OMBB sequences over the major eukaryotic lineages. No TOM40 sequence was identified in *Giardia*, however its presence in this lineage was already reported (Dagley et al. 2009). Tree drawn based on (Burki 2014) and (Mani et al. 2016). LECA: Last Eukaryotic Common Ancestor.

**
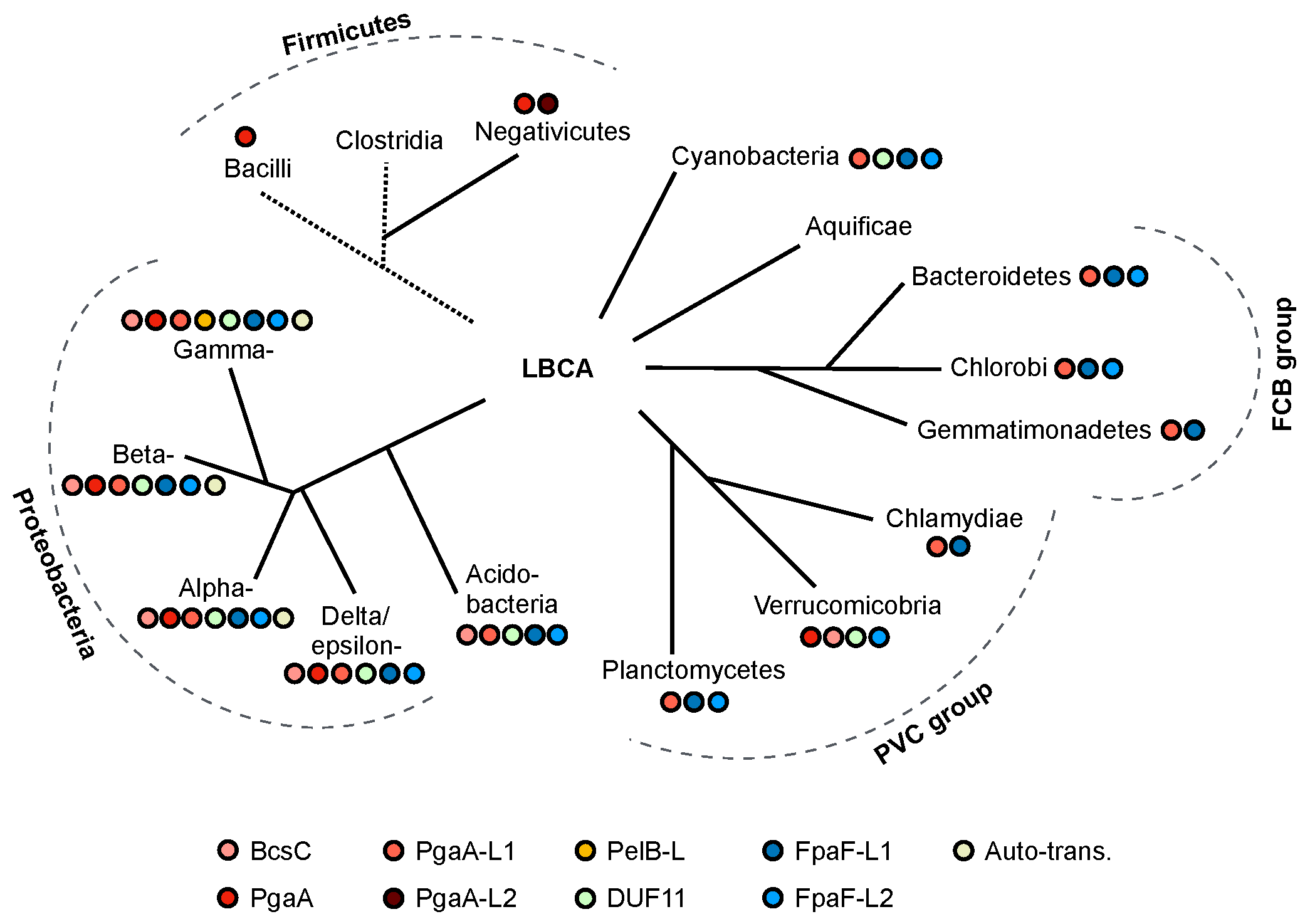
**

Supplementary figure 2. Taxonomic distribution of collected bacterial OMBB sequences over the major Gram-negative and firmicute lineages. A protein family was assigned to a bacterial branch if this branch is represented in more than 0.5% of all sequences for that given protein family. Solid lines represent Gram-negative staining bacteria, while dotted lines illustrate those staining Gram-positive. Tree drawn based on (Hug et al. 2016). LBCA: Last Bacteria Common Ancestor.


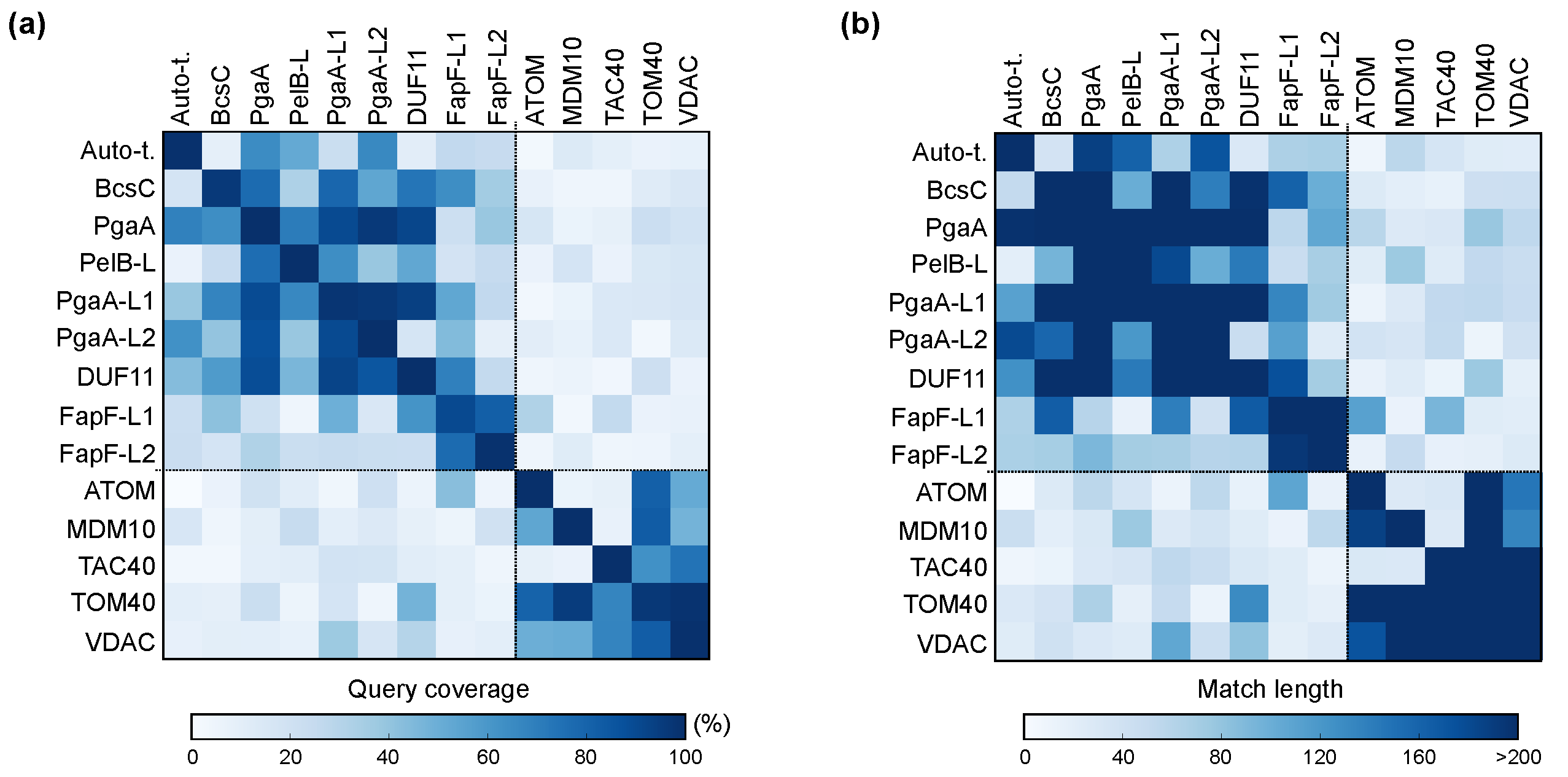


Supplementary figure 3. The length of HMM-comparison matches between bacterial and mitochondrial OMBBs. (a) Relative query coverage matrix and (b) absolute length matrix of the matched regions.

**SUPPLEMENTARY TABLES**

**Supplementary table 1.** Reference sequences and structures used to build the three-dimensional models of MDM10, ATOM, TAC40 and SAM50 with SWISS-PROT. Reference structures were identified with HHPred over the PDB70 without scoring for secondary structure.

| Protein Family | UniProtKB | Template PDBid | Template protein family | Modelled region | Seq. ID (%) | hhsearch probability (%) |
| --- | --- | --- | --- | --- | --- | --- |
| MDM10 | MDM10_YEAST | 5O8O_A | TOM40 | 4-493 | 11.9 | 99.4 |
| ATOM | Tb09.211.1240 | 5O8O_A | TOM40 | 6-351 | 13.2 | 96.4 |
| TAC40 | Tb927.4.1610 | 4C69_A | VDAC | 1-358 | 14.8 | 97.7 |
| SAM50 | SAM50_HUMAN | 4K3B_A | BamA | 1-468 | 19.2 | 99.8 |

**Supplementary table 2.** Matches to OMBBs obtained by HHsearches with the consensus sequences of VDAC and TOM40 double ββ-hairpins over the Pfam, TIGRFAMs, NCBI Conserved Domains (CD) and COG/KOG databases. Searches were preformed with HHPred without secondary structure scoring and only bacterial matches with a probability above 50% are shown. The number of strands, as of their three-dimensional structure identified with HHPred or predicted with Quick2D, and the taxonomic distribution as annotated in EggNOG and Pfam databases, are shown. A family was considered to be present in a given proteobacteria lineage if more than 2 sequences were assigned to it. ββ: ββ-hairpin; n.a.: not applicable.

| *Query* | | *Match* | | | | | | | | | | | | |
| --- | --- | --- | --- | --- | --- | --- | --- | --- | --- | --- | --- | --- | --- | --- |
| Protein family | Repeat unit | OMBB family | ID | Taxonomic distribution in proteobacteria | Reference structure | Biological function | Number of strands | Number of repeats | Repeat unit | Prob. (%) | Number of matches | Number of strands matched | Matched region | Match contains last strand |
| VDAC | 1 | *n.a.* | *n.a.* | *n.a.* | *n.a.* | *n.a.* | *n.a.* | *n.a.* | *n.a.* | *n.a.* | *n.a.* | *n.a.* | *n.a.* | *n.a.* |
| VDAC | 2 | *n.a.* | *n.a.* | *n.a.* | *n.a.* | *n.a.* | *n.a.* | *n.a.* | *n.a.* | *n.a.* | *n.a.* | *n.a.* | *n.a.* | *n.a.* |
| VDAC | 3 | *n.a.* | *n.a.* | *n.a.* | *n.a.* | *n.a.* | *n.a.* | *n.a.* | *n.a.* | *n.a.* | *n.a.* | *n.a.* | *n.a.* | *n.a.* |
| VDAC | 4 | BcsC | PF05420.10 | γ, β, **α**, δ | 4Y25_A | Sugar transp. | 16 | *n.a.* | *n.a.* | 77.0 | 1 | 3 | 288-329 | Yes |
| VDAC | 4 | Ax21 | TIGR04273 | γ | *n.a.* | Quorum-sensing | 10 | 5 | ββ | 72.9 | 1 | 3 | 145-186 | Yes |
| VDAC | 4 | YfaZ | PF07437.10 | γ, β, δ, ε | 3NB3_B | *n.a.* | 8 | *n.a.* | *n.a.* | 67.9 | 1 | 3 | 99-144 | No |
| VDAC | 4 | KdgM | PF06178.12 | γ | 4FQE_A | Sugar transp. | 12 | 6 | ββ | 64.3 | 1 | 3 | 57-103 | No |
| VDAC | 4 | OprF | PF05736.10 | γ, ε | 4RLC_A | Solute transp. | 8 | *n.a.* | *n.a.* | 62.7 | 1 | 3 | 141-184 | Yes |
| VDAC | 4 | Opa | PF02462.14 | β | 1P4T_A | Pathogen-host interaction | 8 | *n.a.* | *n.a.* | 62.1 | 1 | 3 | 78-123 | Yes |
| VDAC | 4 | Porin_7 | PF16956.4 | γ | *n.a.* | *n.a.* | 16 | 8 | ββ | 58.2 | 1 | 4 | 217-267 | Yes |
| VDAC | 4 | myx | TIGR04565 | Oligoflexia | *n.a.* | *n.a.* | 8 | *n.a.* | *n.a.* | 55.3 | 1 | 3 | 110-157 | Yes |
| VDAC | 4 | YaiO | TIGR04390 | γ | *n.a.* | *n.a.* | 14 | *n.a.* | *n.a.* | 53.6 | 1 | 3 | 186-230 | Yes |
| VDAC | 4 | w_GlyGly | TIGR04219 | γ | 1I78_B | *n.a.* | 10 | *n.a.* | *n.a.* | 52.5 | 1 | 3 | 149-191 | No |
| VDAC | 5 | *n.a.* | *n.a.* | *n.a.* | *n.a.* | *n.a.* | *n.a.* | *n.a.* | *n.a.* | *n.a.* | *n.a.* | *n.a.* | *n.a.* | *n.a.* |
| TOM40 | 1 | *n.a.* | *n.a.* | *n.a.* | *n.a.* | *n.a.* | *n.a.* | *n.a.* | *n.a.* | *n.a.* | *n.a.* | *n.a.* | *n.a.* | *n.a.* |
| TOM40 | 2 | *n.a.* | *n.a.* | *n.a.* | *n.a.* | *n.a.* | *n.a.* | *n.a.* | *n.a.* | *n.a.* | *n.a.* | *n.a.* | *n.a.* | *n.a.* |
| TOM40 | 3 | *n.a.* | *n.a.* | *n.a.* | *n.a.* | *n.a.* | *n.a.* | *n.a.* | *n.a.* | *n.a.* | *n.a.* | *n.a.* | *n.a.* | *n.a.* |
| TOM40 | 4 | *n.a.* | *n.a.* | *n.a.* | *n.a.* | *n.a.* | *n.a.* | *n.a.* | *n.a.* | *n.a.* | *n.a.* | *n.a.* | *n.a.* | *n.a.* |
| TOM40 | 5 | *n.a.* | *n.a.* | *n.a.* | *n.a.* | *n.a.* | *n.a.* | *n.a.* | *n.a.* | *n.a.* | *n.a.* | *n.a.* | *n.a.* | *n.a.* |

**REFERENCES**

Burki F. 2014. The eukaryotic tree of life from a global phylogenomic perspective. Cold Spring Harb. Perspect. Biol. 6.

Dagley MJ, Dolezal P, Likic VA, Smid O, Purcell AW, Buchanan SK, Tachezy J, Lithgow T. 2009. The protein import channel in the outer mitosomal membrane of Giardia intestinalis. Mol. Biol. Evol. 26:1941–1947.

Hug LA, Baker BJ, Anantharaman K, Brown CT, Probst AJ, Castelle CJ, Butterfield CN, Hernsdorf AW, Amano Y, Ise K, et al. 2016. A new view of the tree of life. Nat. Microbiol. 1:16048.

Mani J, Meisinger C, Schneider A. 2016. Peeping at TOMs - Diverse entry gates to mitochondria provide insights into the evolution of eukaryotes. Mol. Biol. Evol. 33:337–351.
